# The role of optical coherence tomography guidance in scaffold versus stent optimization

**DOI:** 10.1186/s43044-020-00110-z

**Published:** 2020-11-05

**Authors:** Arif A. Al Nooryani, Nagwa A. Abdelrahman, Hatem A. Helmy, Yehia T. Kishk, Ayman K. M. Hassan

**Affiliations:** 1 Cardiovascular Department, Al Qassimi Hospital, Sharjah, United Arab Emirates; 2 Cardiovascular Department, Faculty of Medicine, Assiut University, Asyut, Egypt

**Keywords:** Bioresorbable scaffold, Optical coherence tomography, Malapposition, Strut fracture

## Abstract

**Background:**

Optical coherence tomography showed a great ability to identify adverse features during percutaneous coronary intervention with drug-eluting stents and resulted in better clinical outcomes. The study aimed to assess the impact of optical coherence tomography on intraoperative decision-making during implantation of Absorb bioresorbable scaffolds versus everolimus drug-eluting stents.

**Results:**

We performed an observational study that included 223 consecutive patients post optical coherence tomography-guided implantation of either Absorb bioresorbable scaffolds (162 patients) or everolimus drug-eluting stents (61 patients). We studied the influence of optical coherence tomography on intraoperative decision-making during implantation of bioresorbable scaffolds versus drug-eluting stents by analyzing the total rate of optical coherence tomography-dependent modifications in each device.

After satisfactory angiographic results, the total rate of required intervention for optical coherence tomography detected complications was significantly higher in the bioresorbable scaffolds arm compared to drug-eluting stents arm (47.8% versus 32.9%, respectively; *p* = 0.019). The additional modifications encompassed further optimization in the case of device underexpansion or struts malapposition, and even stenting in the case of strut fractures, or significant edge dissection.

**Conclusions:**

Compared to drug-eluting stents, Absord scaffold was associated with a significantly higher rate of optical coherence tomography-identified intraprocedural complications necessitating further modifications. The study provides some hints on the reasons of scaffolds failure in current PCI practice; it offers a new insight for the enhancement of BRS safety and presents and adds to the growing literature for successful BRS utilization.

## Background

Despite significant advances in drug-eluting stents (DES) technology, metallic stents are still associated with some drawbacks, as prolonged contact with either the metallic alloy or the polymer accelerates neoatherogenesis with increased hazard of thrombosis and revascularization in addition to vasomotion impairment and physiological blood flow disturbance [9, 11].

Bioresorbable vascular scaffold (BRS) development was a key step in interventional cardiology due to the added advantage of complete scaffold biodegradation, allowing the recovery of vascular pulsatility, vasomotion, and endothelial function compared to DES. The most frequently studied and clinically used scaffold is the Absorb BRS (Abbott Vascular, Santa Clara, California) [8].

Recently, interventional cardiology is at critical crossroads due to the dissatisfactions with the Absorb scaffold. The 3–year follow-up of the ABSORB III trial revealed a higher risk of scaffold thrombosis (ScT). A subsequent systematic review and meta-analysis with > 10,000 patients displayed doubling the risk of ScT [5, 6].

Owing to the inherent differences in recoil characteristics between BRS and DES and the thicker BRS struts, scaffold underexpansion and malapposition are more frequent and are possibly incriminated for the increased risk of scaffold thrombosis [2].

Optical coherence tomography (OCT) showed a great ability to identify adverse features during percutaneous coronary intervention (PCI) with DES, resulting in better clinical outcomes [13].

Recently, the 2018 ESC/EACTS Guidelines on myocardial revascularization upgraded the OCT role due to its accuracy in detecting intraprocedural complications such as malapposition, underexpansion, and edge dissection [12].

In the present study, we aimed to assess the influence of OCT on intraprocedural decision-making during implantation of Absord BRS versus everolimus-DES.

## Methods

### Study design and population

We studied retrospectively consecutive patients (223 patients) who had OCT-guided implantation of either Absorb BRS or everolimus-DES from March 2013 to October 2017 at Al Qassimi Hospital, Sharjah, UAE.

All patients (age ≥ 18 years) received one or more of either Absorb BRS or everolimus-DES, either the Xience everolimus-eluting cobalt–chromium stent (Abbott Vascular, Santa Clara, USA) or the Promus Everolimus-Eluting Platinum-Chromium Stent (Boston Scientific, Massachusetts, USA). Patients who had OCT images with considerable artifacts were excluded from the study.

The influence of OCT guidance on intraoperative decision-making during implantation of BRS versus DES was reported by analyzing the total rate of OCT-based modification in each device.

### BRS implantation in 2013–2017

All patients signed informed consent for scaffold deployment and OCT use before starting the procedure.

All patients were pretreated with aspirin 300 mg and either clopidogrel 600 mg or ticagrelor 180 mg as required.

Lesion predilatation was performed upon operators’ discretion in 231 (94.3%) scaffolds at baseline. Scaffolds were implanted according to common practice with progressive inflation. Post-dilatation with non-compliant (NC) balloon was performed in 235 (96%) scaffolds.

Planned overlapping-BRS strategy was performed when the lesion length exceeded the maximum scaffold length (28 mm), while unplanned overlap was done in the case of significant edge dissection or residual lesion post-scaffold deployment.

### Data collection schedule

An identification number was generated for each patient in an electronic worksheet. Patient’s data (demographics, biological parameters) and coronary angiography review including procedure and lesion description according to the ACC/AHA classification were recorded.

Each stent/scaffold was reviewed to detect if additional intervention was required based on the OCT analysis after what was supposed to be an angiographically successful implantation.

### OCT performance and analysis

OCT acquisition was done using either the ILUMIEN OPTIS PCI Optimization System with the Dragonfly-Duo imaging catheter (both St. Jude Medical, MN, USA) or the LUNAWAVE OCT System with the Fastview imaging catheter (both Terumo, Japan). A non-occlusive technique with injection of iso-osmolar iodixanol (Visipaque) was used for acquisition to limit blood artifacts.

**Fig. 1:**
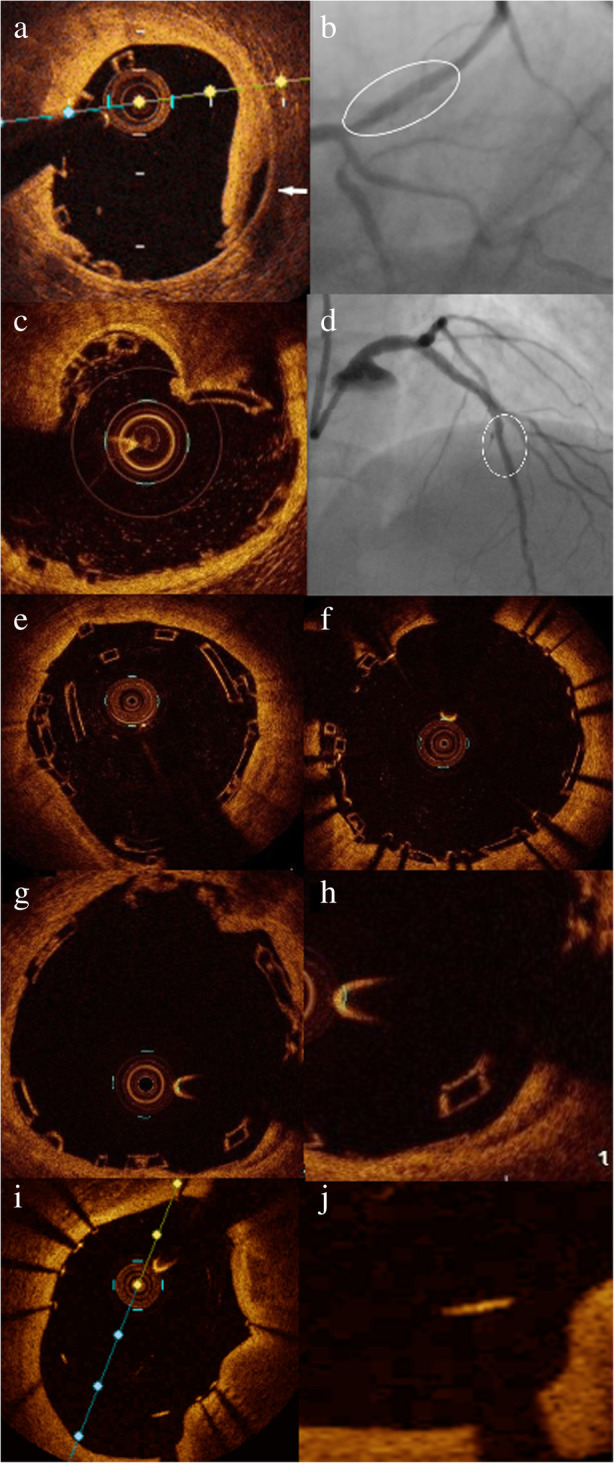
OCT detected BRS and DES complications. **a** OCT image of intimal dissection at the edge of Absorb scaffold (arrow), **b** PA caudal view of the same scaffold implanted in proximal LAD (oval shape) in which edge dissection could not be identified, **c** OCT image of calcium spike inducing scaffold fracture with loss of the expected scaffold circularity, **d** PA cranial view of the same scaffold implanted in mid LAD (oval shape) without evidence of fracture, **e** OCT image of scaffold fracture in the form of overriding contiguous struts post culotte bifurcation stenting, **f** post successful stenting of the same scaffold using DES, **g** OCT showing malapposed scaffold struts, **h** magnified picture showing scaffold struts clearly separated from the vessel wall, **i** OCT picture showing metallic struts malapposition with posterior dropout, and **j** magnified picture showing that the axial distance between the strut’s surface and the luminal surface exceeds the strut thickness in the malapposing struts

Underexpansion was defined as a minimum device area < 80% of the mean proximal and distal reference lumen area [2]. Edge dissection was defined as luminal surface disruption at the stent edge resulting in a flap (Fig. 1a). Residual disease was considered in the case of ≥ 50% narrowing of the mean luminal area in the OCT images within 5 mm proximal or distal to the stent/scaffold edges. Stent fracture was assumed in the case of overriding contiguous struts, disconnection from the expected device circularity, and isolated struts lying unapposed in the lumen (Fig. 1c, e). BRS strut malapposition was defined as the presence of struts separated from the adjacent vessel wall (Fig. 1g, h). In the case of DES inducing posterior dropout, malapposition was considered when the axial distance between the strut’s surface and the luminal surface exceeds the strut thickness (90 um) (Fig. 1i, j) [10].

### Study endpoint

The study endpoint was the total rate of required modifications based on OCT findings, including further post-dilation for underexpansion and malapposition, and additional BRS/DES implantation in the case of significant edge dissection or strut fracture.

### Statistical analysis

Data analysis was done using SPSS version 19 (Statistical Package for Social Science). Data were presented as number, percentage, mean, median, and standard deviation. Chi-square test and Fisher’s exact test were used to compare qualitative variables. Mann-Whitney test was used to compare quantitative variables in case of non-parametric data. A univariate regression analysis was used to identify the independent predictors of the requirement for OCT-based modifications in each arm and to estimate odds ratio (OR) and 95% confidence intervals (CIs). A multivariate logistic analysis was used to identify the independent predictors of OCT-induced PCI modifications in all studied population. First, the following covariates were screened in univariate models: (1) clinical presentation, (2) angiographic variables (target vessel, lesion complexity), and (3) procedural variables (stent type, scaffold/stent diameter). Second, a multivariable analysis of predictors at *p* < 0.20 by univariate analysis was performed to identify independent predictors of OCT-induced PCI modifications and to estimate adjusted odds ratios and 95% confidence intervals (CIs). *p* value < 0.05.was considered statistically significant.

## Results

### Patients and procedures

Consecutive patients (*n*:242) post OCT-guided implantation of either Absorb BRS or everolimus-DES from March 2013 to October 2017 were assessed for eligibility. Nineteen patients were excluded because of artifacts rendering proper OCT images analysis unfeasible. We included 223 patients, with either Absorb BRS (162 patients; 75% males; mean age 53.5 ± 26 years) or everolimus-DES (61 patients; 77% males; mean age 51 ± 25 years).

The Absorb arm had 179 lesions (245 scaffolds), versus 65 lesions (82 stents) in the DES arm.

Patients’ baseline characteristics and PCI indications were almost similar in both groups (Table 1). Lesion and procedural characteristics are summarized in Table 2.

**Table 1:** Baseline demographics and clinical characteristics

Characteristic	Absorb scaffold (***N*** = 162)	Everolimus-DES (***N*** = 61)	***p*** value
	No. (%)	No. (%)	
**Male**	122(75.3%)	47(77.0%)	0.787
**Age/**years	54.36 ± 10.02	54.13 ± 10.89	0.805
**Body mass index**	28.93 ± 4.17	28.97 ± 5.32	0.361
**Hypertension**	104(64.2%)	35(57.4%)	0.349
**Diabetes mellitus**	93(57.4%)	41(67.2%)	0.183
**Insulin-treated**	29(17.9%)	15(24.6%)	0.263
**Dyslipidemia**	120(74.1%)	46(75.4%)	0.838
**Current smoker**	57(36.5%)	23(37.7%)	0.873
**Previous myocardial infarction**	25(15.4%)	10(16.4%)	0.860
**Post-CABG**	2(1.2%)	3(4.9%)	0.127
**Clinical presentation:**			
Silent ischemia	19(11.7%)	7(11.5%)	0.958
Stable angina	50(30.9%)	21(34.4%)	0.611
Unstable angina/NSTEMI	63(38.9%)	28(45.9%)	0.928
Primary PCI	20(12.3%)	4(6.6%)	0.214
Late or lysed STEMI	10(6.2%)	1(1.6%)	0.297

Plus–minus values are means ± SD

*BMI* body mass index is the weight in kilograms divided by the square of the height in meters, *CABG* coronary artery bypass grafting, *DES* drug-eluting stents, *PCI* percutaneous coronary intervention, *STEMI* ST-elevation myocardial infarction

**Table 2:** Lesions and procedural characteristics

Variable	Lesion based		
	Absorb scaffold (***N*** = 179)	Everolimus-DES (***N*** = 65)	***p*** value
**Target vessel:**			
LM	4 (2.2%)	15 (23.1%)	< 0.001
LAD	100 (55.9%)	40 (61.5%)	0.428
LCX	35 (19.6%)	12 (18.4%)	0.863
RCA	43 (24.0%)	10 (15.4%)	0.148
SVG	1 (0.6%)	1 (1.5%)	0.463
**ACC/AHA lesion class B2/C**	116 (64.8%)	46 (70.8%)	0.383
	**Scaffold/stent based**		
	**Absorb scaffold (*n*** **= 245)**	**Everolimus-DES (*n*** **= 82)**	***p*** **value**
**Bifurcation stenting**	18 (7.3%)	17 (20.7%)	< 0.001
**Moderate/severe calcification**	86 (35.1%)	38 (46.3%)	0.069
**Thrombus**	24 (9.8%)	5 (6.1%)	0.308
**Overlap**	141 (57.6%)	59 (72.0%)	0.020
**In-stent implantation**	15 (6.1%)	28 (34.1%)	< 0.001
**Chronic total occlusion**	11 (4.5%)	3 (3.7%)	0.74
**Pre-dilatation**	231 (94.3%)	71 (86.6%)	0.023
**Pre-dilatation NC-balloon**	161 (65.7%)	37 (45.1%)	0.001
**Pre-dilatation cutting balloon**	24 (9.8%)	19 (23.2%)	0.002
**Pre-dilatation balloon/device diameter-ratio**	0.96 ± 0.11	0.90 ± 0.13	< 0.001
**Pre-dilatation inflation pressure/atm**	14.38 ± 3.92	13.79 ± 3.64	0.464
**Stent inflation pressure/atm**	11.95 ± 2.67	12.70 ± 2.87	0.099
**Stent inflation time/sec**	76.66 ± 18.00	16.50 ± 5.57	< 0.001
**Post-dilatation NC-balloon**	235(95.9%)	81 (98.8%)	0.303
**Post-dilatation balloon/device diameter ratio**	1.10 ± 0.09	1.08 ± 0.12	0.025
**Post-dilatation balloon-device diameter/mm**	0.34 ± 0.25	0.29 ± 0.34	0.04
**Post-dilatation inflation pressure/atm**	17.89 ± 4.04	16.84 ± 4.03	0.028
**Post-dilatation inflation time/sec**	15.88 ± 6.22	13.70 ± 3.44	0.004
**Device success**	239 (97.6%)	81 (98.8%)	0.685
**Use of bailout device**	25 (10.2%)	1 (1.2%)	0.009

Plus–minus values are means ± SD

*ACC/AHA* American College of Cardiology/American Heart Association, *DES* drug-eluting stents, *LM* left main, *LAD* left anterior descending, *LCX* left circumflex, *NC* non-compliant, *RCA* right coronary artery, *SVG* saphenous vein graft

### OCT-detected intraoperative complications

After satisfactory angiographic results, the total rate of required intervention for OCT-detected complications was significantly higher in the BRS arm compared to the DES arm (47.8% versus 32.9%, respectively; *p* = 0.019), (Table 3).

**Table 3:** Comparison of OCT-guided modification in BRS vs. DES

Modifications	BRS (***n*** = 245)	DES (***n*** = 82)	***p*** value
	No. (%)	No. (%)	
**Total modifications**	117 (47.6)	27 (32.9)	0.019
**Post-dilatation due to underexpansion or malapposition**	90 (36.7)	26 (31.7)	0.410
**Dissection stenting**	16 (6.5)	1 (1.2)	0.060
**Stenting due to underexpansion**	2 (0.8)	0 (0.0)	0.411
**Fracture stenting**	4 (1.6)	0 (0.0)	0.576
**Thrombus aspiration**	1 (0.4)	0 (0.0)	0.562
**Thrombus stenting**	1 (0.4)	0 (0.0)	0.562
**Residual lesion stenting**	3 (1.2)	0 (0.0)	0.576

*BRS* bioresorbable scaffolds, *DES* drug-eluting stent

Stenting for OCT-detected dissection was required in 6.5% of BRS and 1.2% of DES arms (*p* = 0.06) (Fig. 1a, b), (video 1 and 2 in the supplemental material). Underexpansion necessitating stenting was observed in 2 scaffolds (0.8%) that showed significant underexpansion despite multiple attempts of postdilatation with NC balloon at high pressure, so upgrade of the NC balloon size and pressure beyond the manufacturer limit was required, and thus stents were implanted for fear of struts fracture. Stenting due to fractured struts was required in 4 scaffolds (1.6%), but not in the DES arm (Fig. 1c–f). Thrombus aspiration post BRS implantation was attempted by the operators in a case where thrombus was extending to more than 3 frames (6 mm), appearing as a thrombus protruding between struts in one frame (8 × 6 mm), and as small thrombi moving freely inside the lumen in other frames. Thrombus stenting was done in a case of a large thrombus (4 × 3 mm) protruding out of the distal scaffold edge with a free tail in the vessel lumen, which could be a substrate for scaffold thrombosis, or at least microvascular occlusion and no-reflow. The total rate of bailout devices used was significantly higher in the BRS arm (*n*:25; 10.2%) compared to DES arm (*n*:1; 1.2%) (*p* = 0.009).

Among 6 cases with acute scaffold failure, two were due to heavily calcified lesions with subsequent underexpansion, as well as OCT-detected struts fracture in 4 scaffolds. All 6 lesions were treated successfully with DES (Fig. 1c–f). There were no adverse events associated with OCT imaging in the studied population.

NSTEMI presentation, type B2/C lesion, moderate/severe calcification, and overlapping stents, all were found to have a significant positive relationship with the need for intraoperative modification post-OCT review in the BRS arm. Bifurcation stenting was the only variable that has a positive relation in the DES arm (Table 4).

**Table 4:** Univariate logistic regression analysis of OCT based modification in BRS and DES

	BRS		DES	
	Odds ratio (95% CI)	***p*** value	Odds ratio (95% CI)	***p*** value
**Clinical presentation:**				
CSA	1.77 (0.9–3.48)	0.095	1.02 (0.34–3.04)	0.964
UA	1.34 (0.54–3.31)	0.519	0.99 (0.21–4.59)	0.99
NSTEMI	2.165 (1.03–4.52)	0.04*	0.42 (0.13–1.39)	0.158
Late/lysed MI	0.7 (1.19–2.59)	0.59	–	–
Primary PCI	2.2 (0.82–5.84)	0.113	1.71 (0.22–13.08)	0.603
Silent ischemia	0.45 (0.16–1.26)	0.131	5.0 (0.88–28.33)	0.069
**Target vessel:**				
LAD	1.03 (0.57–1.87)	0.911	2.18 (0.7–6.72)	0.175
LCX	0.56 (0.25–1.21)	0.144	0.76 (0.172–3.38)	0.721
RCA	1.62 (0.815–3.24)	0.168	0.63 (0.14–2.72)	0.539
**ACC/AHA B2/C**	1.82 (1.016–3.28)	0.044*	1.96 (0.63–6.07)	0.239
**Bifurcation stenting**	1.5 (0.57–3.95)	0.407	2.93 (0.981–8.79)	0.05*
**Moderate/severe calcification**	2.41 (1.407–4.15)	0.001*	1.30 (0.55–3.5)	0.484
**Overlap**	1.84 (1.09–3.08)	0.021*	1.56 (0.53–4.57)	0.413
**In-stent restenosis**	1.81 (0.62–5.27)	0.272	1.9 (0.74–5.07)	0.171
**CTO**	0.65 (0.18–2.29)	0.509	1.01 (0.08–11.76)	0.988
**Ostial**	0.65 (0.32–1.31)	0.234	1.39 (0.55–3.52)	0.484

*ACC/AHA* American College of Cardiology/American Heart Association, *BRS* bioresorbable scaffold, *CSA* chronic stable angina, *CI* confidence interval, *CTO* chronic total occlusion, *DES* drug-eluting stents, *LAD* left anterior descending, *LCX* left circumflex, *NSTEMI* non-ST elevation myocardial infarction, *OCT* optical coherence tomography, *PCI* percutaneous coronary intervention, *RCA* right coronary artery, *UA* unstable angina

By performing a multivariate logistic regression analysis of all studied population, BRS scaffold, type B2/C lesion, moderate/severe calcification, and primary PCI presentation, all were found to have a significant relationship with the OCT-detected complications post device implantation (Table 5).

**Table 5:** Multivariate logistic regression analysis of OCT-based modification in the studied population

	Univariate		Multivariate	
	Odds ratio (95% CI)	***p*** value	Odds ratio (95% CI)	***p*** value
**Stent type (BRS)**	1.74 (1.03–2.94)	0.038*	2.35 (1.28–4.31)	0.006*
**Clinical presentation:**				
CSA	0.76 (0.48–1.22)	0.271	–	–
UA	0.79 (0.42–1.47)	0.459	–	–
NSTEMI	2.02 (1.19–3.42)	0.009*	1.71 (0.97–3.01)	0.062
Late/Lysed MI	1.8 (0.67–4.82)	0.238	–	–
Primary PCI	2.51 (1.15–5.46)	0.02*	2.55 (1.1–5.9)	0.028*
Silent ischemia	0.96 (0.49–1.89)	0.917	–	–
**Target vessel:**				
LM	0.52 (0.2–1.34)	0.177	0.6 (0.18–1.93)	0.397
LAD	0.87 (0.56–1.37)	0.57	–	–
LCX	1.85 (0.96–3.55)	0.064	1.34 (0.64–2.82)	0.431
RCA	0.67 (0.41–1.11)	0.124	0.74 (0.41–1.34)	0.327
**ACC/AHA B2/ C lesion class**	1.84 (1.1–3.09)	0.02*	1.78 (1.02–3.11)	0.039*
**Bifurcation stenting**	0.59 (0.29–1.2)	0.15	0.52 (0.22–1.21)	0.131
**Moderate/severe calcification**	1.88 (1.19–2.95)	0.006*	1.99 (1.2–3.3)	0.029*
**Overlap**	1.64 (1.04–2.6)	0.03*	1.39 (0.82–2.33)	0.214
**In-stent restenosis**	0.75 (0.39–1.43)	0.39	–	–
**CTO**	1.36 (0.44–4.16)	0.585	–	–
**Ostial lesion**	1.34 (0.79–2.28)	0.275	–	–
**Stent diameter**	1.09 (0.68–1.75)	0.714	–	–

*ACC/AHA* American College of Cardiology/American Heart Association, *BRS* bioresorbable scaffold, *CSA* chronic stable angina, *CI* confidence interval, *CTO* chronic total occlusion, *LAD* left anterior descending, *LCX* left circumflex, *NSTEMI* non-ST elevation myocardial infarction, *OCT* optical coherence tomography, *PCI* percutaneous coronary intervention, *RCA* right coronary artery, *UA* unstable angina

## Discussion

The invention of bioresorbable vascular scaffolds was considered a revolution in the field of interventional cardiology due to the potential advantages of scaffold bioresorption.

To our knowledge, this is the first study to compare the influence of OCT on the operators’ decisions during deployment of Absorb BRS versus everolimus-eluted metallic stents.

Optimal scaffold placement entails a balance between perfect struts embedment and absence of edge dissection. Uncorrected residual stenosis, struts malapposition, and edge dissections increase the risk of future scaffold restenosis and thrombosis [2].

In the present study, there was a significant difference in the impact of OCT on intraoperative decision-making during implantation of BRS versus DES. Despite angiographic success, we reported a significantly higher rate of OCT detected device-related complications in the BRS arm (47.8%) compared to the DES arm (32.9%).

The detected complications necessitated further modifications such as further optimization in case of device underexpansion or struts malapposition, and even additional stenting in case of strut fractures, or significant edge dissection reaching up to 10.2% of bailout device use in the BRS arm versus 1.2% only in the DES arm.

In ABSORB cohort B substudy, OCT detected suboptimal deployment in more than 25% of patients with mainly type A lesions, and this number might have theoretically increased with the expanded BRS use into complex lesions [1, 14]. This theory was verified by our findings, where type B2/C lesion, moderate/severe lesion calcification, and overlapping stents had a positive relationship with the need for OCT-guided additional interventions, emphasizing the importance of lesion selection before BRS implantation. BRS scaffold was found to have a significant positive relationship with the OCT-based intraoperative modification, thus highlighting the value of OCT guidance for BRS optimization.

Even if not identified on angiography, the OCT-detected complications may have a critical impact on patients’ outcome if left untreated [13]. It is well known that the recent Absorb setback was mainly due to a higher tendency of very late scaffold thrombosis in the ABSORB trials [5, 15].

Increased rates of subacute scaffold thrombosis in ABSORB III trial were explained by the high rate of residual stenosis, while the high very late scaffold thrombosis rate in ABSORB II was attributed to underexpansion and incomplete coverage due to malapposition, as detected with intravascular ultrasound (IVUS) [3, 4]. In the ABSORB Japan trial, OCT showed incomplete coverage due to overhanging struts in very late scaffold thrombosis cases as malapposing struts prevent scaffold bioresorption and delay endothelialization, thus increasing the risk of thrombosis [7].

The difference in the rate of required interventions between both devices can be explained by the intrinsic biomechanical differences due to the eccentric expansion pattern of the Absorb scaffold that results into a higher rate of underexpansion and struts malapposition [2]. Moreover, the radiolucency of the scaffold to the conventional angiography renders underexpansion and fractures less likely to be spotted by this modality.

A significant difference in the implantation technique adopted by the operators during BRS versus DES deployment was noted, as post-dilatation balloon/device diameter ratio, inflation time, and pressure were higher in the BRS arm (1.10 ± 0.09 vs 1.08 ± 0.12, *p* = 0.025; 15.88 ± 6.22 s vs13.70 ± 3.44 s, *p* = 0.004; and 17.89 ± 4.04 atm vs 16.84 ± 4.03 atm, *p* = 0.028, respectively), most likely governed by OCT findings of malapposition and underexpansion.

OCT displayed intraoperative complications requiring further interventions in 32.9% of DES arm, in concordance with the CLI-OPCI study that reported a 34.7% rate of additional interventions based on OCT findings during PCI using metallic stents [13]. The current results highlight the value of OCT-guidance also during DES implantation.

### Study limitations

The study has some limitations, including those typical of non-randomized studies; however, the retrospective design has some advantages in the present study, as it prevented the operators’ bias while studying the impact of OCT on intraprocedural operators’ behavior. Additionally, substantial discrepancies between groups could not be avoided due to the retrospective time-limited study design; nevertheless, despite higher lesions complexity in the DES group, a significantly lower rate of modifications was required and thus strengthens the study results; however, we should not forget that no method can accurately adjust for all known and unknown confounders. Besides, any malapposition not considering axial and/or longitudinal distance (axial distance > 300 μm and longitudinal extension > 1.0 mm, not associated with side branches) may lead to the overestimation of complications detected by OCT after device implantation because minor malapposition (< 0.35 mm) will be resolved with neointima at follow-up. Finally, despite that the intraprocedural OCT assessment was verified by a different team during the study, the lack of corelab analysis is considered a limitation.

## Conclusions

Compared to DES, Absorb scaffold was associated with a significant higher rate of OCT-detected intraprocedural complications requiring further modifications. The study provides some hints on the reasons of scaffolds failure in current PCI practice; it offers a new insight for the enhancement of BRS safety and presents and adds to the growing literature for successful BRS utilization.

## Supplementary Information

**Additional file 1:** Video 1. Coronary angiography of PA caudal view showing ABSORB scaffold implanted in proximal LAD segment, without evidence of proximal edge dissection, linked to Fig. 1b.

**Additional file 2:** Video 2. OCT run in proximal segment of same scaffold in proximal LAD, showing proximal edge dissection as evident by intimal disruption at the scaffold edge, linked to Fig. 1a.

## Data Availability

Raw data will be available upon request by the editorial board.

## Abbreviations

BRS: Bioresorbable scaffold(s)
DES: Drug-eluting stent(s)
MI: Myocardial infarction
NC balloon: Non-compliant balloon
OCT: Optical coherence tomography
PCI: Percutaneous coronary intervention
ScT: Scaffold thrombosis
STEMI: ST-segment elevation myocardial infarction
